# Is Pulse Co-Oximetry a Reliable Alternative to Invasive Hemoglobin Measurement in Pediatric Neurosurgical Procedures?

**DOI:** 10.3390/children13030323

**Published:** 2026-02-25

**Authors:** Funda Arun, Oguzhan Arun

**Affiliations:** 1 Department of Pedodontics, Division of General Anesthesia, Faculty of Dentistry, Selcuk University, Selcuklu, Konya 42130, Türkiye; funda.arun@selcuk.edu.tr; 2 Department of Anesthesiology and Reanimation, Faculty of Medicine, Selcuk University, Alaaddin Keykubat Campus, Akademi Mahallesi Yeni Istanbul Caddesi No:313, Selcuklu, Konya 42130, Türkiye

**Keywords:** SpHb, pediatric neurosurgery, non-invasive monitoring, hemoglobin, anemia, pulse CO-Oximetry, perfusion index, metabolic acidosis, hemodynamic instability, patient blood management

## Abstract

**Highlights:**

**What are the main findings?**

• Non-invasive hemoglobin monitoring systematically overestimates laboratory values, with error margins significantly widening during severe metabolic acidosis (Base Excess < −10 mEq/L).
• Hemodynamic autoregulation is compromised during hypotensive episodes, creating a pressure-dependent perfusion state that degrades measurement precision even when the Perfusion Index appears clinically normal.

**What are the implications of the main findings?**

• Clinicians should prioritize SpHb for continuous trend monitoring rather than as a sole trigger for transfusion, particularly in patients with hemodynamic or metabolic instability.
• Since “normal” perfusion index values can persist during hypotension and mask instability, non-invasive monitoring must be cross-validated with invasive sampling and metabolic markers.

**Abstract:**

**Background/Objectives:** Pediatric neurosurgical procedures often involve significant blood loss and rapid hemodynamic shifts, necessitating accurate hemoglobin (Hb) monitoring. While continuous non-invasive Hb (SpHb) monitoring offers real-time trending, its accuracy in high-risk pediatric populations remains debated. We aimed to evaluate the diagnostic accuracy and clinical utility of SpHb compared to invasive arterial blood gas (ABG) analysis in pediatric patients undergoing cranial and spinal surgeries. **Methods:** This prospective, observational study enrolled 60 pediatric patients (aged 0–16 years) scheduled for high-risk neurosurgery. SpHb was measured continuously and compared with intermittent ABG-Hb values. Statistical analysis included Bland–Altman agreement, Pearson’s correlation, and Error Grid Analysis. Subgroup analyses assessed the impact of the Perfusion Index (PI), hypotension, and metabolic acidosis on device performance. **Results:** Data from 57 patients (median age: 12 months, interquartile range: 6–42 months; 70.2% aged <24 months) were analyzed. SpHb demonstrated a moderate correlation with ABG-Hb (r = 0.567, *p* < 0.001) but exhibited systematic overestimation with a mean bias of +1.60 ± 1.54 g/dL. Crucially, SpHb showed 0% sensitivity for detecting critical anemia (Hb < 8.0 g/dL). Device performance was significantly compromised by physiological extremes: severe metabolic acidosis significantly increased bias to +2.27 g/dL (*p* = 0.038), and intraoperative hypotension significantly widened the limits of agreement (SD of bias: 1.79 g/dL vs. 1.45 g/dL in normotension). Furthermore, hemodynamic analysis revealed a loss of autoregulation during hypotension, where the pressure-perfusion coupling strengthened (r = 0.44) compared to the normotensive state (r = 0.15). **Conclusions:** SpHb monitoring provides fair Hb trending but is limited by systematic overestimation and poor sensitivity for critical anemia. Accuracy worsens during severe acidosis and hemodynamic instability. Therefore, SpHb should function as a complementary “early warning” trend monitor rather than a sole transfusion trigger, with invasive validation remaining essential for intraoperative decision-making.

## 1. Introduction

Pediatric neurosurgical procedures carry a substantial risk of significant blood loss and anemia, critically influencing outcomes. This susceptibility is driven by surgical complexity, low body weight, and preoperative hemoglobin (Hb) reserves [1]. Managing these complications presents a dilemma, as both anemia and allogeneic transfusions are independent predictors of adverse events. Anemia-induced hypoxia triggers oxidative stress, neuroinflammation, and endothelial dysfunction, impairing neurovascular coupling. Specifically, the overproduction of reactive oxygen species disrupts blood–brain barrier integrity, exacerbating neuronal injury [2]. Conversely, transfusions carry risks of inflammatory reactions, thrombosis, and circulatory overload, potentially worsening brain injury [3]. Given the pediatric population’s narrower tolerance for anemia, suboptimal management increases morbidity, mortality, and costs [4]. Therefore, accurate, real-time intraoperative Hb monitoring is paramount to guide transfusion decisions and mitigate risks.

Although laboratory analysis remains the gold standard for Hb assessment, its utility is constrained by invasiveness, intermittent sampling delays, and the cumulative risk of iatrogenic anemia-factors particularly concerning in pediatric patients [5]. To address these limitations, continuous noninvasive Hb (SpHb) monitoring has emerged as a promising clinical tool. Pulse CO-oximetry technology utilizes multi-wavelength spectrophotometry to measure Hb levels via a finger probe, analogous to standard pulse oximetry [6]. By providing real-time trend data without the pain or infection risk of frequent phlebotomy, this technology holds the potential to support clinical decision-making across a broad spectrum of environments, ranging from high-acuity settings like the operating room and intensive care unit to routine screening in outpatient clinics.

Extensive research has investigated the accuracy and clinical utility of SpHb monitoring via pulse co-oximetry across the pediatric spectrum. The literature encompasses diverse clinical scenarios, ranging from emergency trauma assessment and the management of solid organ injuries to critical care settings, including the neonatal and pediatric intensive care units, particularly in the evaluation of patients with shock [7,8]. Furthermore, the application of this technology has expanded beyond intraoperative trending to functional perioperative uses, such as non-invasive preoperative anemia screening [9,10], fluid kinetic modeling [11], and postoperative surveillance in orthopedic wards [12].

However, despite the promising findings, the current literature is characterized by significant limitations. In the majority of studies, baseline Hb values remain well above critical thresholds or clinical intervention limits. Perioperative research has predominantly focused on children with low surgical risk profiles undergoing procedures where significant hemorrhage is unanticipated. Even in studies involving surgeries with potential for bleeding, the volume of blood loss is frequently insufficient to induce hemodynamic instability or reduce Hb to critical levels. Furthermore, although the pediatric spectrum spans from birth to 18 years, most studies have been restricted to narrow age cohorts, failing to provide a comprehensive evaluation across all developmental stages. This scarcity of data is starkly highlighted by a recent systematic review, which identified that among 39 eligible studies evaluating SpHb monitoring, only two were exclusively dedicated to the pediatric population [13]. Moreover, from a methodological standpoint, reliance on traditional statistical metrics such as bias and precision has been criticized for potentially masking inaccuracies in the critical lower Hb ranges where transfusion decisions are made, prompting calls for more clinically relevant evaluation methods like error grid analysis [14,15].

Considering the scarcity of pediatric-specific data in the current literature, this study aims to evaluate the diagnostic accuracy and clinical utility of SpHb across a comprehensive pediatric age spectrum-ranging from neonates to adolescents-undergoing high-risk neurosurgical procedures (cranial and spinal) characterized by significant potential for blood loss.

## 2. Methods

### 2.1. Study Design and Patient Population

This single-center, prospective, observational study was conducted in the Department of Neurosurgery at Selcuk University Faculty of Medicine, following the approval of the Local Ethics Committee (E-70632468-050.01.04-271261), and registration at ClinicalTrials.gov (NCT07352033). Sixty pediatric patients, aged 0–16 years and classified according to the American Society of Anesthesiologists (ASA) physical status classification I-IV, scheduled for elective complex spinal surgery or cranial procedures under general anesthesia, were enrolled after obtaining written parental informed consent. The study population consisted of cases where significant blood loss was anticipated. This anticipation was strictly based on predefined high-risk procedural categories (e.g., open cranial vault reconstruction, craniosynostosis repair, and complex spinal surgery) according to institutional protocols that mandate routine invasive arterial blood pressure monitoring, rather than subjective real-time surgeon judgment. Exclusion criteria included congenital heart disease, requirement for emergency surgery, and pre-existing peripheral circulatory disorders. Additionally, patients with a history of hemoglobinopathies, severe hyperbilirubinemia, methemoglobinemia, recent intravenous dye administration (within 24 h), or those with local factors interfering with SpHb sensor accuracy—such as severe edema or nail abnormalities (e.g., nail polish, henna) at the application site—were excluded from the study.

### 2.2. General Anesthesia Protocol

Upon arrival at the operating suite, all patients—having adhered to ASA fasting guidelines and received no premedication—underwent standard ASA monitoring, including electrocardiography (ECG), pulse oximetry (SpO_2_), and non-invasive blood pressure (NIBP) measurement. During the monitoring phase, midazolam (0.05–0.1 mg/kg) was administered to patients with existing intravenous (IV) access. In these patients, anesthesia was induced with 1 mg/kg lidocaine, 2–3 mg/kg propofol, 0.6 mg/kg rocuronium, and 1.5 µg/kg fentanyl. For patients lacking pre-existing IV access, inhalation induction was initiated with 8% sevoflurane in 100% oxygen. Following the loss of consciousness, the sevoflurane concentration was gradually titrated downward to maintain hemodynamic stability. Once intravenous (IV) access was secured, midazolam (0.05–0.1 mg/kg) and a bolus of 1–2 mg/kg propofol were administered immediately to deepen anesthesia. Following this, 1 mg/kg lidocaine, 1.5 µg/kg fentanyl, and 0.6 mg/kg rocuronium were administered to complete the induction sequence. Concurrently, the sevoflurane concentration was adjusted to 3% to facilitate tracheal intubation with an appropriately sized cuffed or uncuffed endotracheal tube. Following the securing of the airway via tracheal intubation, continuous IABP monitoring was established in all patients. For arterial cannulation, the radial artery was the primary site of choice in older children; however, in neonates and small infants, the femoral artery was preferred due to its larger vessel caliber and greater technical accessibility in this age group. Given the complex nature of these surgical procedures and the potential for significant intraoperative blood loss, central venous cannulation was performed in all patients. The subclavian or femoral vein was selected as the insertion site, based on the patient’s age, anatomical considerations, and the specific surgical field. Anesthesia was maintained either with 2–3% sevoflurane or via a Total Intravenous Anesthesia (TIVA) protocol consisting of propofol (6–10 mg/kg/h) and remifentanil (0.1–0.5 µg/kg/min) infusions in a 50:50 oxygen–air mixture. In both groups, anesthetic delivery and infusion rates were dynamically titrated to maintain BIS values within the target clinical range of 40 to 60 and to ensure hemodynamic stability throughout the surgical procedure.

Intraoperative fluid management was performed using a suitable crystalloid solution, with the maintenance rate calculated via the Holiday–Segar (4-2-1) formula. The preoperative fluid deficit was replaced over three hours: 50% was administered in the first hour, followed by 25% in the second hour, and the remaining 25% in the third hour. Core body temperature was monitored nasopharyngeally, with a clinical target of maintaining normothermia (36.0–37.5 °C). Active warming was provided using a forced-air system (Bair Hugger; 3M, St. Paul, MN, USA) from the time of surgical positioning until emergence. Concurrently, axillary skin temperature was monitored on the extremity equipped with the SpHb sensor to ensure adequate peripheral perfusion and minimize measurement inaccuracies in non-invasive Hb readings.

### 2.3. Data Collection and Laboratory Analysis

For each patient enrolled in the study, a comprehensive set of clinical variables was systematically documented and evaluated. Demographic and baseline characteristics included age, gender, height, weight, and body mass index (BMI). Preoperative clinical data comprised the ASA physical status scores, the presence of comorbidities and medications, and the duration of preoperative fasting. Intraoperative and surgical parameters were recorded in detail, encompassing the specific type of surgical procedure, the total duration of the operation, and the duration of anesthesia. Furthermore, perioperative operational data were documented, including the estimated volume of blood loss, the administration of blood or blood products, the total amount of intravenous fluids administered, and the requirement for vasoactive drug support.

The primary focus of this study was the continuous monitoring of SpHb through non-invasive Pulse CO-Oximetry technology. For this purpose, a Masimo Radical-7 Pulse CO-Oximeter (Masimo Corp., Irvine, CA, USA) was utilized for all measurements. To ensure optimal signal acquisition and measurement accuracy across the pediatric population, two types of patient-specific sensors were employed based on body weight: RD rainbow SET-2 Infant sensors were used for patients weighing between 3 kg and 30 kg, while RD rainbow SET-2 Neo (Neonatal/Adult) sensors were preferred for patients weighing less than 3 kg or more than 30 kg, in accordance with the manufacturer’s clinical specifications. An appropriately sized adhesive multi-wavelength sensor was applied to the nail bed of a suitable finger or, in infants, the great toe. To ensure the highest signal quality and minimize measurement bias, the SpHb sensor was consistently placed on an extremity distal to and contralateral to the site of invasive arterial monitoring. For patients with radial artery cannulation, the sensor was applied either to a finger of the opposite hand or to the great toe of an infant, depending on which site provided the most optimal Perfusion Index (PI). Similarly, for patients with femoral artery cannulation, the sensor was positioned on a finger of the upper extremity or the contralateral great toe. This strategic positioning was maintained to prevent potential interference from arterial flow dynamics, localized vasomotor changes, or intermittent limb occlusion during blood pressure measurements on the cannulated side. Measurement was initiated immediately upon the patient’s arrival in the operating room and continued throughout the procedure. To ensure high-quality signal acquisition, the sensor was shielded from ambient light, and the extremity was monitored for adequate perfusion via the PI. SpHb values, along with SpO_2_ and PI, were captured and recorded digitally at the exact time points when arterial blood gas (ABG) samples were drawn. ABG analyses were performed using a calibrated Siemens RAPIDLab 1200 System (Siemens Healthineers, Erlangen, Germany). In addition to Hb and oxygenation, the ABG analysis included the evaluation of key physiological parameters such as pH, partial pressure of arterial carbon dioxide (PaCO_2_), lactate, and base excess (BE) to monitor the patient’s metabolic and acid-base status comprehensively. At the conclusion of the surgical procedure, a final ABG analysis was performed; simultaneously, a separate blood sample was collected and sent for a venous complete blood count (CBC) to serve as the reference laboratory standard. These synchronized intraoperative measurements were subsequently compared with the CBC and ABG results for clinical validation.

### 2.4. Statistical Analysis

The study’s sample size was determined based on the total number of paired data sets required to evaluate the agreement and correlation between non-invasive SpHb and laboratory Hb measurements. To ensure a statistical power of 0.80 with an alpha level of 0.05, a target of at least 200 paired observations was established, allowing for robust sub-group analyses across diverse intraoperative and hemodynamic conditions. To achieve this data volume while accounting for potential technical exclusions and clinical variability in the number of blood samples obtained per procedure, the enrollment of 60 patients was targeted. The primary unit of analysis was defined as the individual paired data sets. Given the observational nature of the intraoperative dynamic changes, paired data sets were treated as independent observations without explicit statistical adjustment for within-patient clustering.

Statistical analyses were performed using IBM SPSS Statistics v26.0 and Python 3.10.12 and SciPy 1.11.4 library. The normality of the data distribution was assessed using the Shapiro–Wilk test. Descriptive statistics were expressed as mean ± standard deviation (SD) for normally distributed variables and as median (interquartile range) for non-normally distributed data. The agreement between non-invasive SpHb and reference ABG-Hb was evaluated using Bland–Altman analysis, calculating the mean bias and the 95% limits of agreement (LoA = 1.96\times SD). Proportional bias was investigated using linear regression. Pearson’s correlation coefficient (r) was used to assess the strength of the linear relationship. The trending ability of SpHb was assessed via Four-Quadrant Plot analysis. The concordance rate was calculated by excluding Hb changes < 0.5 g/dL to minimize statistical noise. Clinical performance was further evaluated by calculating sensitivity and specificity at clinical decision thresholds of 8.0 g/dL and 9.0 g/dL. Subgroup analyses were conducted to determine the impact of physiological variables on device performance. Measurements were stratified based on PI categories derived from manufacturer recommendations (<0.3, 0.3–1.0, 1.0–5.0, and >5.0), hemodynamic status with hypotension (defined as MAP < 45 mmHg for infants (<12 months), < 55 mmHg for children 1–5 years, and <65 mmHg for those >5 years), preoperative fasting duration, and baseline Hb levels. Furthermore, the influence of metabolic status (defined by BE < −10 mEq/L), crystalloid fluid intensity (stratified by cohort median), and transfusion timing (pre- vs. post-transfusion) were evaluated to assess device stability under dynamic clinical conditions.

## 3. Results

Of the 60 patients initially enrolled, 3 were excluded due to technical difficulties during data collection. Consequently, the final analysis was conducted on the remaining 57 patients, encompassing a total of 210 individual evaluations.

### 3.1. Patient Characteristics

The study population’s demographic characteristics are summarized in Table 1. The cohort (n = 57) was predominantly male (n = 41, 71.9%; female: n = 16, 28.1%), with a mean age of 33.2 ± 43.8 months (median: 12; range: 1–168 months). Mean weight and height were 13.9 ± 10.2 kg (range: 3.3–58.0 kg) and 87.5 ± 28.8 cm (range: 50.0–165.0 cm), respectively. Patients were categorized into two groups for growth assessment:

• Infants and Toddlers (<24 months, n = 40, 70.2%): Evaluated by weight and length, the group showed a mean weight of 8.8 ± 2.3 kg (range: 3.3–15.0 kg) and a mean length of 71.4 ± 9.5 cm (range: 50.0–95.0 cm).
• Children ≥ 24 months (n = 17, 29.8%): Evaluated by BMI, the group had a mean BMI of 15.8 ± 3.0 kg/m^2^ (range: 9.4–21.3 kg/m^2^).

Nutritional status was defined as significantly underweight if the BMI was below 15 kg/m^2^ (for patients ≥ 24 months) or if the weight-for-age was below −2 SD per WHO growth charts (for patients < 24 months). Under these criteria, 12 patients (21.1%) were identified as significantly underweight (n = 7 in the older group; n = 5 in the younger group).

### 3.2. Surgical and Perioperative Data

The study population’s surgical and perioperative data are summarized in Table 1. According to the ASA physical status classification, patients were distributed as follows: ASA I (n = 11, 19.3%), ASA II (n = 28, 49.1%), ASA III (n = 17, 29.8%), and ASA IV (n = 1, 1.8%). While 77.2% (n = 44) had no additional medical history, 22.8% (n = 13) presented with at least one comorbidity, including hydrocephalus (n = 4), spina bifida (n = 2), laryngomalacia (n = 1), hypospadias (n = 2), retinal coloboma (n = 1), hydronephrosis (n = 2), hemangioma (n = 2), Apert syndrome (n = 1), and Asperger syndrome (n = 1). Some patients contributed multiple conditions to this count. Both prior surgical history and chronic medication use were documented in 14.0% (n = 8) of the cohort. The mean preoperative fasting duration was 6.8 ± 1.5 h (median: 6.0; range: 4.0–11.0 h). Adherence to the 2-4-6 rule (4–6 h window) was 64.9% (n = 37), while 84.2% (n = 48) were compliant with the standard 6–8 h elective hospital window.

Craniosynostosis repair (including specific head shapes such as scaphocephaly and trigonocephaly) was the most common intervention (52.6%, n = 30), followed by intracranial mass/hemorrhage surgeries (24.6%, n = 14). Other procedures included kyphectomy (n = 4, 7.0%), and calvarial mass excision (5.3%, n = 3). The remaining cases consisted of cranioplasty (n = 2), hemangioma excision (n = 1), craniectomy (n = 1), and scoliosis surgery (n = 1). The mean operative time was 198.6 ± 66.5 min (median: 200; range: 60–400), with most procedures (interquartile range) completed between 160 and 225 min.

Intraoperative bleeding averaged 147.6 ± 192.6 mL (range: 10–1000 mL), corresponding to 12.7 ± 15.4 mL/kg or 15.9% of estimated total blood volume (EBV). Significant bleeding (≥10% of EBV) occurred in 59.6% (n = 34) of patients. Transfusion was required in 70.2% (n = 40) for erythrocyte suspension (mean: 11.41 ± 4.6 mL/kg) and 33.3% (n = 19) for fresh frozen plasma (mean: 12.06 ± 4.2 mL/kg). Mean crystalloid administration was 22.74 ± 13.01 mL/kg (median: 19.02; range: 2.50–61.11 mL/kg).

### 3.3. Analysis of Hemoglobin Value Trends and Consistency

**Figure 1:**
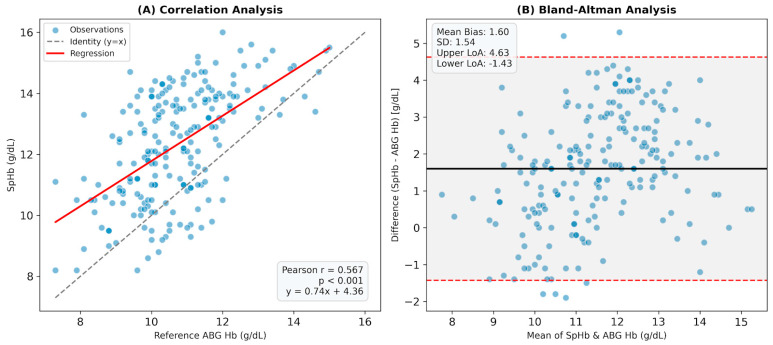
Agreement and correlation analysis between SpHb and reference ABG Hb. (**A**) Correlation Analysis: The scatter plot demonstrates a moderate positive correlation (r = 0.567, *p* < 0.001). The solid red line represents the linear regression fit, while the dashed gray line indicates the line of identity (y = x). (**B**) Bland–Altman Analysis: The plot illustrates the agreement between the two methods. The solid black line indicates the systematic overestimation (Mean Bias: +1.60 g/dL). The dashed red lines represent the 95% LoA (Upper: +4.63 g/dL, Lower: −1.43 g/dL), with the shaded area representing the precision range.

The relationship between SpHb and the reference ABG Hb was evaluated using Pearson’s correlation, revealing a moderate, significant positive correlation (r = 0.567, *p* < 0.001). Bland–Altman analysis showed a mean bias of 1.60 ± 1.54 g/dL, indicating SpHb tends to overestimate Hb levels. The 95% limits of agreement (LoA) ranged from −1.43 to +4.63 g/dL (Figure 1).

**Figure 2:**
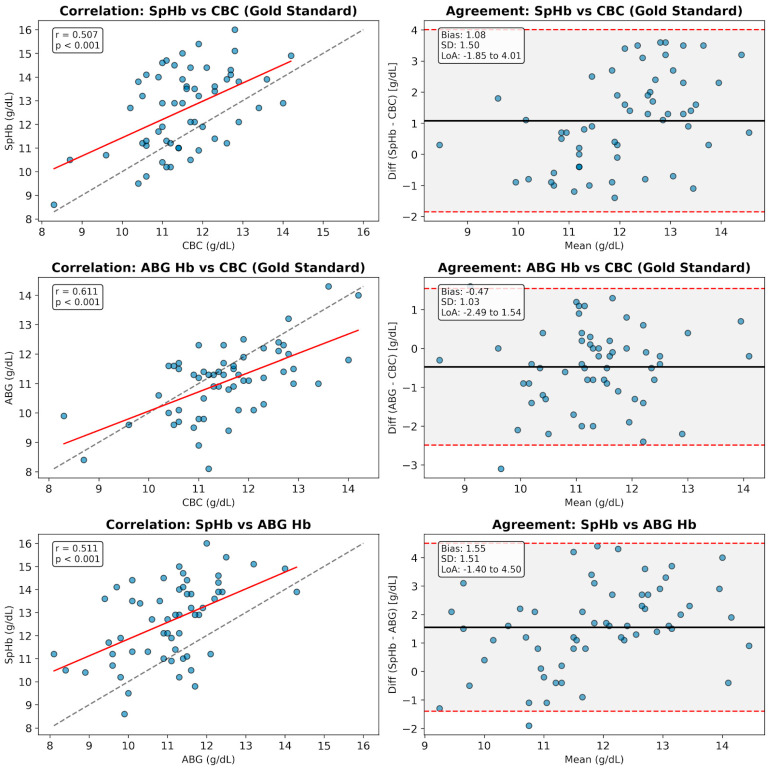
Comparison of SpHb and ABG Hb against the laboratory gold standard (CBC) at the conclusion of surgery. The matrix displays scatter plots with regression analysis (Left Column) and Bland–Altman agreement plots (Right Column) for three paired comparisons: (Top Row) SpHb vs. CBC: SpHb demonstrated a moderate correlation (r = 0.507) but systematically overestimated Hb levels with a mean bias of +1.08 g/dL. (Middle Row) ABG Hb vs. CBC: ABG analysis showed the strongest correlation (r = 0.611) and the highest accuracy, with a minimal negative bias of −0.47 g/dL. (Bottom Row) SpHb vs. ABG Hb: The device showed a similar correlation pattern (r = 0.511) and overestimation bias (+1.55 g/dL) against the point-of-care ABG reference. Solid red lines represent the linear regression (left) and mean bias (right), while dashed lines indicate the 95% LoA.

At the conclusion of surgery, final SpHb, ABG Hb, and CBC Hb measurements were significantly correlated (*p* < 0.001). ABG Hb showed the strongest correlation with the CBC gold standard (r = 0.598), followed by SpHb (r = 0.476); the SpHb-ABG correlation was r = 0.500. Bland–Altman analysis revealed that SpHb systematically overestimated Hb compared to CBC (bias: +1.05 g/dL) and ABG (bias: +1.60 g/dL), whereas ABG Hb slightly underestimated CBC levels (bias: −0.54 g/dL) (Figure 2). Despite similar trends across all parameters, ABG Hb demonstrated superior accuracy and closer agreement with laboratory results at the end of the procedure.

**Figure 3:**
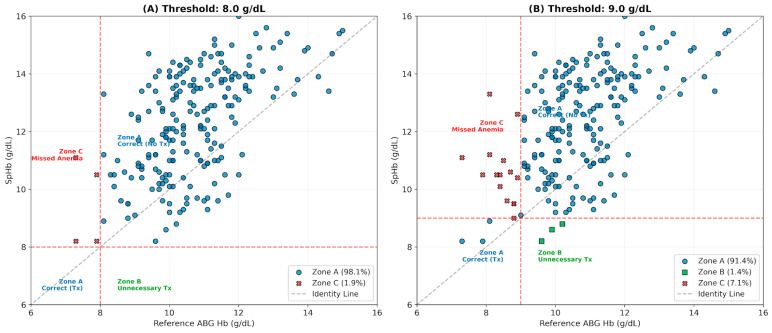
Comparative Hemoglobin Error Grid analysis at standard and early-warning thresholds. The figure displays the clinical decision zones for SpHb measurements using two different transfusion (Tx) triggers. (**A**) Standard Threshold (8.0 g/dL): At this critical limit, the device showed high specificity with 98.1% of measurements in Zone A (Correct Decision). However, 1.9% of cases fell into Zone C (Missed Anemia), representing false negatives. (**B**) Early Warning Threshold (9.0 g/dL): Increasing the threshold to 9.0 g/dL resulted in a higher rate of “Missed Anemia” (Zone C increased to 7.1%), as the device’s systematic overestimation caused more anemic patients (Hb < 9.0 g/dL) to be misclassified as safe (SpHb > 9.0 g/dL). Zone A (Blue): Correct Clinical Decision; Zone B (Green): Unnecessary Transfusion; Zone C (Red): Missed Anemia. The increase in Zone C in Panel B illustrates that while raising the threshold captures more potential cases, it also exposes the limitations of the device’s accuracy near the decision boundary. The dashed red lines represent the clinical decision thresholds (8.0 g/dL in Panel (**A**) and 9.0 g/dL in Panel (**B**)) used to delineate the error grid zones.

Using a transfusion threshold of 8.0 g/dL, the Hemoglobin Error Grid showed that 98.1% of measurements fell within Zone A (Correct Clinical Decision). Only 0.95% of data points were in Zone C (Missed Anemia), where SpHb remained above the threshold despite ABG Hb falling below (Figure 3A). Absolute accuracy was limited: 28.1% of readings were within 1.0 g/dL and 43.8% within 1.5 g/dL of the reference. While SpHb demonstrated 100% specificity for identifying patients not requiring transfusion, it failed to detect any of the four instances below 8.0 g/dL (these events occurred across 3 unique patients; 0% sensitivity, exact binomial 95% CI: 0.0% to 60.2%).

The potential of SpHb as an early warning tool for anemia was evaluated by comparing the 8.0 g/dL transfusion trigger with a 9.0 g/dL “warning” threshold. At 8.0 g/dL, SpHb showed 0% sensitivity, failing to detect all 4 reference events. Increasing the threshold to 9.0 g/dL improved sensitivity to 16.7% (identifying 3 of 18 events). Specificity remained high at both levels (100% at 8.0 g/dL; 98.4% at 9.0 g/dL), effectively ruling out anemia (Figure 3B).

**Figure 4:**
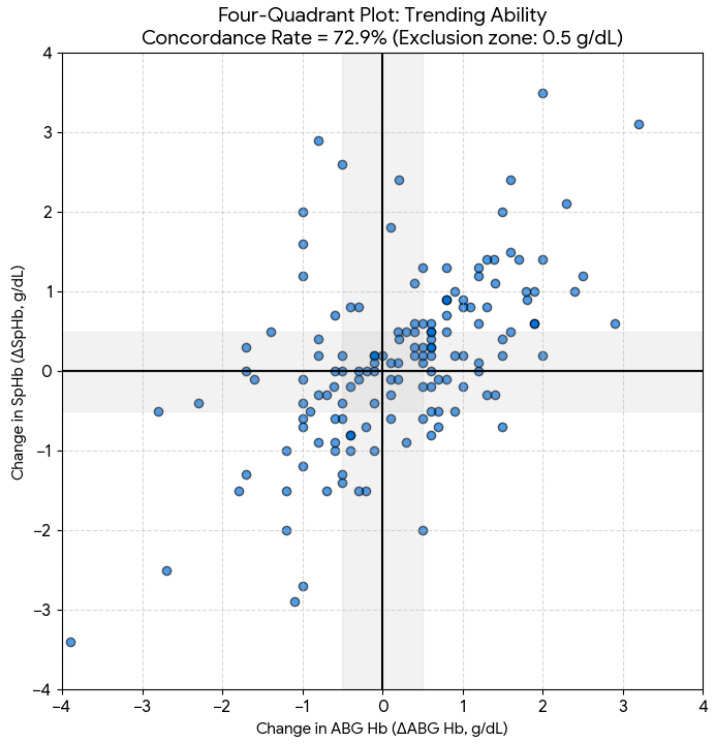
Four-Quadrant Plot of Trending Ability. The graph shows the directional agreement between changes in ABG Hb (ΔABG Hb) and changes in SpHb (ΔSpHb). The calculated concordance rate of 72.9% reflects the device’s ability to track hemoglobin trends over time. The shaded central region represents the 0.5 g/dL exclusion zone, which was applied to filter out clinically insignificant changes and measurement noise.

The dynamic tracking capability of SpHb was evaluated using a Four-Quadrant Plot on 154 consecutive measurement pairs (derived from 210 observations after excluding baseline points and pairs with missing subsequent data). Applying a 0.5 g/dL exclusion zone to ABG Hb changes resulted in an overall concordance rate of 72.9% (n = 118) (Figure 4). While SpHb correctly identified the direction of Hb changes in nearly three-quarters of instances, this falls below the >90% threshold typically required for clinical reliability in trend-monitoring.

**Figure 5:**
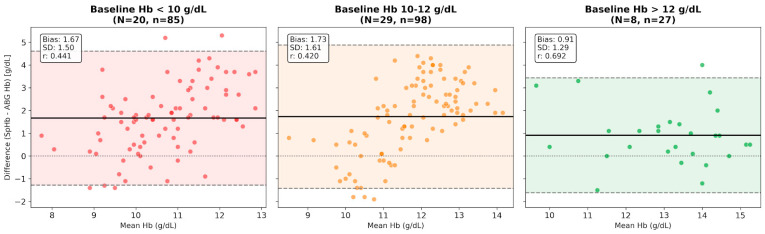
Bland–Altman analysis stratified by baseline Hb levels. The panels display the agreement between SpHb and ABG Hb, categorized by the patient’s initial intraoperative Hb (T1). (Left and Middle Panels) In patients with low (<10 g/dL) or intermediate (10–12 g/dL) baseline Hb, SpHb exhibited a significant systematic overestimation, with mean bias increasing to +1.67 g/dL and +1.73 g/dL, respectively. (Right Panel) In patients starting with normal Hb levels (>12 g/dL), the device demonstrated superior accuracy (lower bias: +0.91 g/dL) and stronger correlation. The solid black line represents the mean bias, while the dashed lines indicate the 95% LoA. This trend suggests that SpHb accuracy is “Hb-dependent,” with performance degrading in patients who are already anemic or near-anemic at the start of surgery. The colored shaded regions represent the 95% limits of agreement (precision range) for each respective baseline hemoglobin subgroup.

To assess SpHb reliability relative to baseline physiological states, measurements were stratified by the initial ABG Hb (T_1_). In patients starting with normal levels (>12 g/dL; N = 10, n = 31), SpHb showed the strongest correlation (r = 0.682, *p* < 0.001) and highest accuracy (mean bias: +0.75 g/dL). Conversely, systematic overestimation was significantly higher in patients entering surgery with lower levels: a bias of +1.74 g/dL for the 10–12 g/dL group (N = 27, n = 94, *p* = 0.008) and +1.67 g/dL for the <10 g/dL group (N = 19, n = 85, *p* = 0.014) (Figure 5).

To evaluate the impact of perfusion on SpHb performance, patients were stratified into four PI categories. Correlation between SpHb and ABG Hb was significant in all groups except for the low-perfusion category (PI < 0.3, n = 11; r = 0.477, *p* = 0.138). In contrast, significant correlations were maintained across the remaining groups: 0.3 ≤ PI < 1 (r = 0.637, *p* < 0.001), 1 ≤ PI < 5 (r = 0.531, *p* < 0.001), and PI ≥ 5 (r = 0.648, *p* = 0.002). Bland–Altman analysis revealed that mean bias increased alongside perfusion, rising from 0.65 ± 1.70 g/dL in the PI < 0.3 group to 1.82 ± 1.37 g/dL in the PI ≥ 5 group (Table 2).

**Figure 6:**
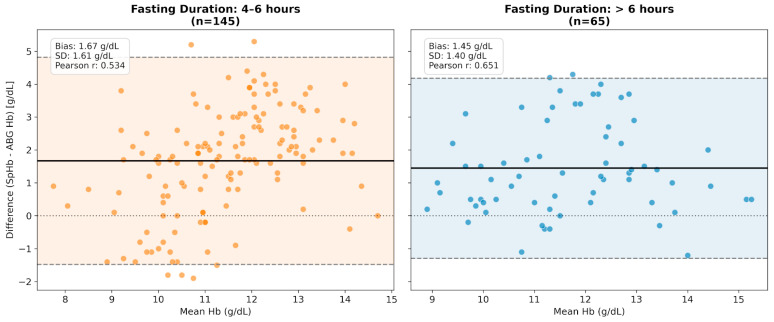
Impact of preoperative fasting duration on SpHb accuracy. The Bland–Altman plots compare device performance between patients with standard fasting duration (4–6 h) and those with prolonged fasting (>6 h). (Left Panel) In the 4–6 h group, the device showed a mean bias of +1.67 g/dL with a precision (SD) of 1.61 g/dL. (Right Panel) Contrary to the dehydration hypothesis, the >6 h group exhibited improved performance, characterized by a lower mean bias (+1.45 g/dL), narrower limits of agreement (SD: 1.40 g/dL), and a stronger correlation (r = 0.651 vs. r = 0.534). The solid black line represents the mean bias, and the shaded regions indicate the 95% LoA. The horizontal dotted line at zero represents the line of perfect agreement (zero bias).

To investigate the effects of preoperative fasting, patients were categorized by fasting duration: 4–6 h vs. >6 h. Both groups maintained significant correlations between SpHb and ABG Hb (*p* < 0.001), though the correlation was stronger in the >6 h group (r = 0.651 vs. r = 0.534). Contrary to expectations that fasting-induced dehydration might degrade performance, the >6 h group showed slightly lower bias (+1.45 g/dL vs. +1.67 g/dL) and improved precision (SD: 1.40 vs. 1.61 g/dL) (Figure 6).

**Figure 7:**
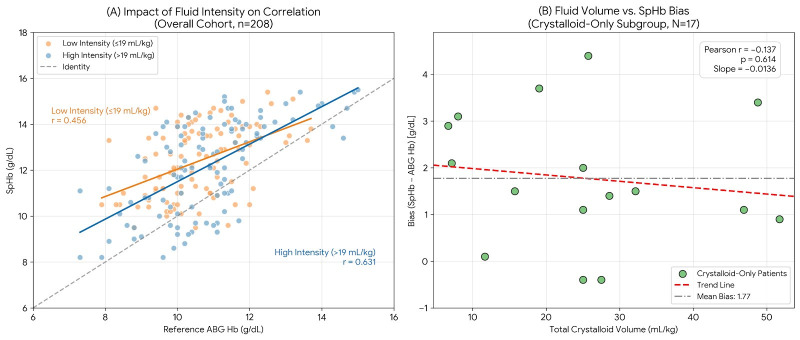
Impact of crystalloid fluid administration on SpHb performance. (**A**) Correlation Analysis in Overall Cohort: The scatter plot compares SpHb-ABG correlation between patients receiving lower (≤19 mL/kg) versus higher (>19 mL/kg) crystalloid volumes. Contrary to concerns about hemodilution artifacts, the high-intensity group demonstrated a stronger correlation (r = 0.631) compared to the low-intensity group (r = 0.456). (**B**) Volume-Bias Relationship in Crystalloid-Only Subgroup: In patients who received exclusively crystalloids (no blood products), there was no significant correlation between weight-adjusted fluid volume and measurement bias (r = −0.137). The trend line (dashed red) remains flat, indicating that increasing crystalloid hemodilution does not independently exacerbate the systematic overestimation bias. The solid orange and blue lines represent the linear regression trends for the low-intensity and high-intensity crystalloid groups, respectively.

The influence of crystalloid intensity on SpHb performance was evaluated across the cohort and in a refined subgroup. In the overall cohort (n = 208), higher fluid intensity (defined as >19.0 mL/kg, the cohort median) did not degrade performance; instead, SpHb demonstrated a stronger correlation (r = 0.631) compared to the lower intensity group (r = 0.456), though this difference did not reach statistical significance (*p* = 0.075). To further isolate the effect of peak hemodilution without the confounding influence of blood products, a subgroup analysis was conducted on patients receiving only crystalloids (N = 17). At the conclusion of surgery, the mean bias remained nearly identical between the low and high fluid groups (defined as >25 mL/kg, the cohort median) (+1.79 g/dL vs. +1.76 g/dL; *p* > 0.99). No significant correlation was found between weight-adjusted crystalloid volume and SpHb bias (r = −0.136, *p* = 0.60) (Figure 7).

**Figure 8:**
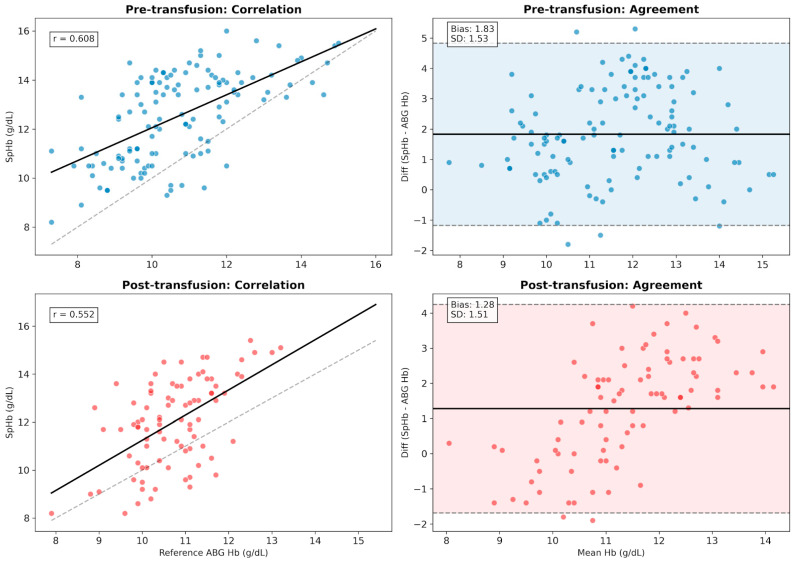
Impact of erythrocyte suspension (ES) transfusion on SpHb performance. The figure compares the correlation and agreement of SpHb before and after blood transfusion. (Top Row) Pre-transfusion State (n = 123): In the period preceding transfusion (including non-transfused patients), SpHb showed a robust correlation (r = 0.608) with a mean bias of +1.83 g/dL. (Bottom Row) Post-transfusion State (n = 87): Following ES administration, while the mean bias mathematically narrowed to +1.28 g/dL (likely due to higher Hb levels), the correlation strength decreased (r = 0.552). Interpretation: The degradation in correlation despite improved bias suggests a “lag effect,” where the device’s trend-tracking ability is slightly attenuated during the acute hematological shifts associated with transfusion. In the correlation plots (left column), the solid black lines represent the linear regression fit, and the dashed gray lines indicate the line of identity. In the Bland-Altman plots (right column), the solid black lines represent the mean bias, the dashed lines indicate the 95% limits of agreement (LoA), and the shaded areas (shadows) illustrate the precision range.

Measurements were categorized based on the timing of ES administration during the procedure. The pre-transfusion group (n = 123) included all samples from non-transfused patients and samples taken before the start of transfusion in the ES group. The post-transfusion group (n = 87) comprised measurements obtained after ES was administered. In the pre-transfusion state, SpHb showed a robust correlation (r = 0.608) with a mean bias of +1.83 ± 1.53 g/dL. Following transfusion, the correlation decreased to r = 0.552 (*p* < 0.001), while the mean bias narrowed to +1.28 ± 1.51 g/dL (*p* = 0.011) (Figure 8).

### 3.4. Hematological and Hemodynamic Profile

Baseline Hb levels, obtained via CBC one day before surgery, averaged 12.07 ± 1.45 g/dL (median: 12.00; range: 8.60–15.60 g/dL). Among the cohort, 12 patients (21%) presented with preoperative anemia (Hb < 11.0 g/dL). The mean Hb level recorded at the conclusion of surgery (wound closure) was 11.58 ± 1.19 g/dL (range: 8.30–14.80 g/dL), representing the final intraoperative hematological status. Following neurosurgical literature defining postoperative anemia as Hb < 10.0 g/dL, only 3 patients (5.3%) were identified as anemic. The majority (94.7%) maintained levels above this threshold, while 5 patients (8.8%) exceeded 13.0 g/dL. Intraoperative hemodynamic stability was assessed across 210 paired observations using age-specific mean arterial pressure (MAP) targets: 45 mmHg for infants (<12 months), 55 mmHg for children 1–5 years, and 65 mmHg for those >5 years. The overall mean MAP was 59.35 ± 13.83 mmHg (range: 28–119 mmHg), with normotension maintained in 73.3% (154/210) of measurements.

Age-specific MAP distribution:

• Infants (n = 106): Mean MAP was 53.29 ± 13.93 mmHg; hypotension was observed in 28.3% of measurements.
• Children 1–5 years (n = 62): Mean MAP was 63.45 ± 11.13 mmHg; 19.4% incidence of hypotension.
• Children >5 years (n = 42): Mean MAP was 68.60 ± 9.20 mmHg; 33.3% incidence of hypotension.

**Figure 9:**
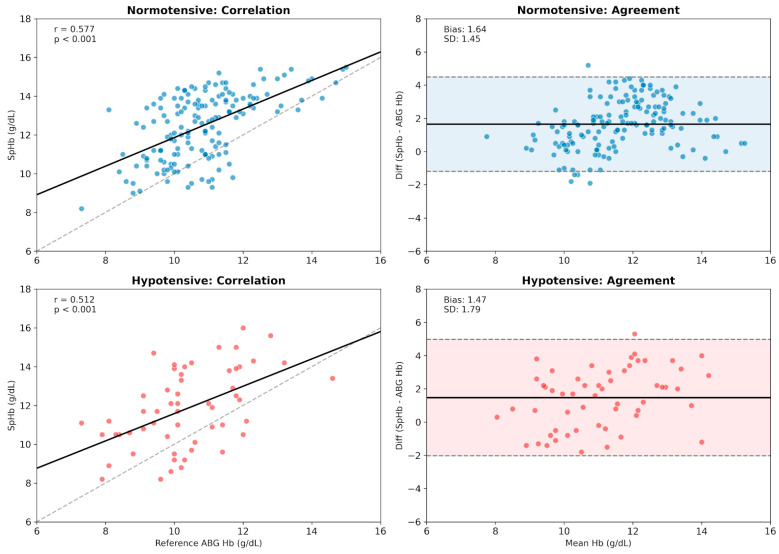
Impact of intraoperative hypotension on SpHb measurement reliability. The composite figure contrasts device performance between normotensive (Top Row) and hypotensive (Bottom Row) conditions. (Left Column—Correlation) While normotensive patients exhibited a stronger correlation (r = 0.577), hemodynamic instability weakened this relationship (r = 0.512), reflecting signal degradation. (Right Column—Agreement) Bland–Altman analysis reveals that hypotension significantly compromises precision. Although the mean bias remained comparable, the limits of agreement widened in the hypotensive group (SD increased from 1.45 to 1.79 g/dL), confirming that low arterial pressure leads to more erratic and unpredictable readings. In the correlation plots (left column), the solid black lines represent the linear regression fit, and the dashed gray lines indicate the line of identity. In the Bland-Altman plots (right column), the solid black lines represent the mean bias, the dashed lines indicate the 95% limits of agreement (LoA), and the shaded areas (shadows) illustrate the precision range.

In the normotensive group (n = 154), SpHb demonstrated a stronger correlation (r = 0.577) and higher precision (SD of bias: 1.45). Conversely, the hypotensive group (n = 56) exhibited a weaker correlation (r = 0.512) and significantly lower precision (SD of bias: 1.79). While the mean bias was slightly lower during hypotension (+1.47 g/dL vs. +1.64 g/dL), the increased SD indicates more erratic and less predictable measurements (Figure 9). Although the systematic bias remained similar between groups (*p* = 0.52), hypotension significantly degraded measurement precision (Levene’s test, *p* = 0.02), leading to wider limits of agreement and less reliable trend tracking.

**Figure 10:**
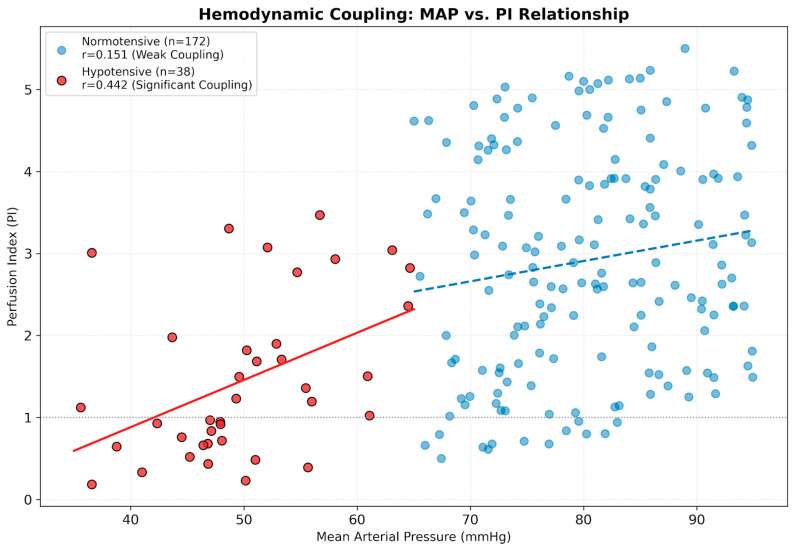
Loss of hemodynamic autoregulation during hypotension. The scatter plot contrasts the relationship between Mean Arterial Pressure (MAP) and Perfusion Index (PI) across the entire dataset of 210 measurement points. Normotensive (Blue, n = 172): The weak correlation (r = 0.15) indicates intact autoregulation, where local perfusion is maintained independently of systemic pressure fluctuations. Hypotensive (Red, n = 38): The stronger correlation (r = 0.44, *p* = 0.006) suggests a pressure-dependent state, where the autoregulatory buffer is exhausted and local perfusion becomes coupled to systemic pressure. The blue dashed line and the red solid line represent the linear regression trends for the normotensive and hypotensive groups, respectively.

The relationship between systemic pressure and local perfusion was evaluated across the entire cohort (n = 210) to assess hemodynamic coupling. In the normotensive state (n = 172), PI remained robust (mean: 3.08 ± 1.24); only 3.5% of measurements fell below 1.0, and remarkably, no cases (0%) of critical hypoperfusion (PI < 0.3) were observed, while a weak correlation with MAP (r = 0.15) indicated intact autoregulation. Conversely, this hemodynamic coupling significantly strengthened during hypotensive episodes (n = 38; r = 0.44, *p* = 0.006), suggesting a pressure-dependent perfusion state. Despite this coupling, hypotension did not consistently result in critical ischemia; only 44.7% (n = 17) of hypotensive events coincided with a PI < 1.0, and only 5.3% (n = 2) fell below the manufacturer-recommended threshold of 0.3. In the remaining 55.3% of cases, PI remained normal or high despite systemic hypotension (Figure 10). Nevertheless, the high SD of bias (1.92) observed during these episodes indicates that hemodynamic instability degrades SpHb precision, even when PI values appear clinically ‘reliable’.

**Figure 11:**
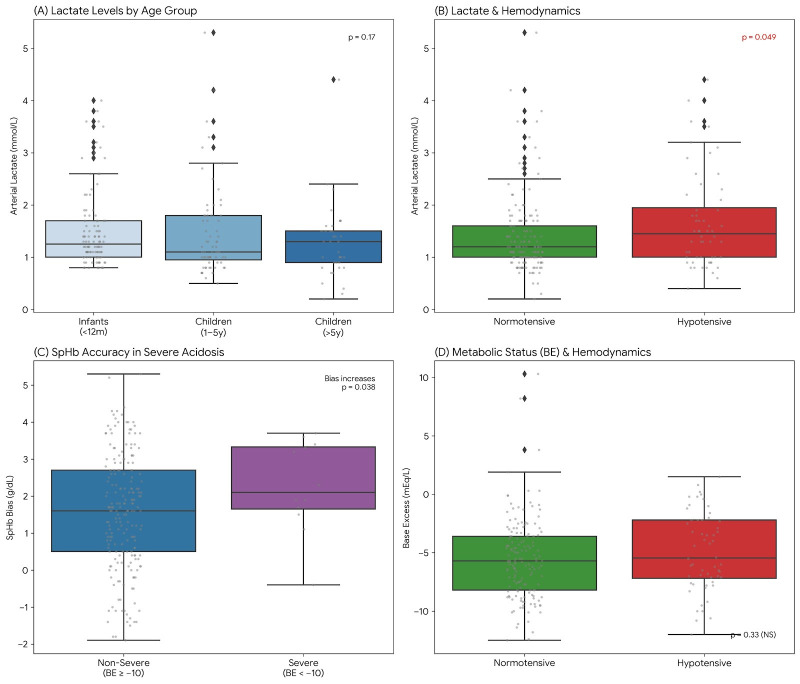
Comprehensive metabolic and perfusion profile during pediatric neurosurgery. (**A**) Arterial lactate levels remained within physiological ranges across all age groups (*p* = 0.17). (**B**) Hypotensive episodes were associated with a modest but statistically significant increase in lactate levels (1.66 vs. 1.39 mmol/L; *p* = 0.049). (**C**) Impact of severe metabolic acidosis (BE < −10 mEq/L) on device performance; mean bias significantly increased from +1.56 to +2.27 g/dL under severe acidotic conditions (*p* = 0.038). (**D**) Base Excess (BE) levels showed no significant difference between normotensive and hypotensive states (*p* = 0.33), indicating that metabolic acidosis was independent of transient blood pressure fluctuations.

Tissue perfusion was monitored via arterial lactate levels, yielding a corrected overall mean of 1.46 ± 0.79 mmol/L across 210 measurement points. Mean lactate values remained within age-appropriate physiological ranges for all groups (*p* = 0.17; Figure 11A): 1.51 ± 0.74 mmol/L in infants (<12 months), 1.48 ± 0.89 mmol/L in children aged 1–5 years, and 1.28 ± 0.73 mmol/L in children >5 years. Perfusion was effectively maintained during normotensive periods (mean lactate: 1.39 ± 0.73 mmol/L), with only a modest increase to 1.66 ± 0.91 mmol/L during age-specific hypotensive episodes (*p* = 0.049; Figure 11B). Longitudinal assessment revealed that 96.5% of patients maintained stable values, confirming that hypotension caused only transient and manageable metabolic shifts.

Acid-base balance monitoring showed a cohort mean Base Excess (BE) of −5.37 ± 3.43 mEq/L. During the procedure, 35% of patients experienced a significant BE decrease (>3 mEq/L), reflecting the metabolic shift typical of major pediatric neurosurgery. While the device correlation generally weakened in unstable patients, the impact of metabolic status was particularly pronounced; in instances of severe metabolic acidosis (BE < −10 mEq/L; n = 12), the mean bias increased significantly from +1.56 g/dL to +2.27 g/dL (*p* = 0.038; Figure 11C). This indicates that the device’s tendency to overestimate Hb levels intensifies under severe acidotic conditions. Importantly, BE levels remained comparable between normotensive (−5.52 ± 3.46 mEq/L) and age-specific hypotensive (−4.93 ± 3.34 mEq/L) states (*p* = 0.33; Figure 11D).

Intraoperative oxygenation remained optimal, with 210 paired measurements showing a mean PaO_2_ of 192.01 46.32 mmHg and SaO_2_ of 99.39% ± 0.91%. No hypoxemia (PaO_2_ < 60 mmHg or SaO_2_ < 94%) occurred. SpO_2_ levels were consistently high via both the Masimo sensor (99.49% ± 1.00%) and standard monitoring (99.19% ± 1.33%). These stable, supranormal oxygenation conditions confirm that hypoxia did not confound SpHb sensor performance.

## 4. Discussion

This study evaluated the intraoperative performance of non-invasive SpHb monitoring in pediatric neurosurgery. Our findings demonstrate that while SpHb provides a fair representation of Hb trends, it exhibits a consistent systematic overestimation—averaging +1.6 g/dL—and a concordance rate (72.9%) that remains below the established threshold for autonomous clinical use. The reliability of SpHb was notably sensitive to the patient’s physiological state; precision and trend-tracking were significantly compromised during periods of low peripheral perfusion, systemic hypotension, and acute Hb shifts following blood transfusion. Conversely, moderate variations in preoperative fasting and crystalloid infusion intensity did not negatively impact sensor performance. Collectively, these results suggest that although SpHb serves as a valuable continuous monitoring adjunct, its tendency to overestimate oxygen-carrying capacity—particularly in already anemic or hemodynamically unstable patients—precludes it from replacing ABG measurements for definitive intraoperative decision-making.

The existing pediatric literature on SpHb monitoring is predominantly characterized by studies conducted in non-operative settings, such as outpatient anemia screening, emergency department trauma triage, or neonatal intensive care units [16,17,18]. For instance, studies by Ryan et al. in pediatric solid organ injuries and Bhat et al. in various clinical scenarios suggested that pulse co-oximetry could serve as a feasible screening tool due to its non-invasive nature and acceptable accuracy in relatively stable patients [19,20]. However, the physiological challenges encountered in these environments differ fundamentally from those in high-risk pediatric surgery. While non-operative studies often involve patients with stable peripheral perfusion, the intraoperative phase introduces confounding factors such as anesthetic-induced vasodilation, mechanical ventilation, rapid fluid shifts, and the type of infused fluids. These variables are known to interfere with spectrophotometric signal quality, suggesting that the clinical reliability of the device must be evaluated specifically within the dynamic and high-stress environment of the operating room rather than through the lens of stable clinical screening.

When focusing specifically on the perioperative literature, many of the limited pediatric studies have evaluated SpHb in cohorts where significant hemorrhage was unanticipated or the surgical risk profile was relatively low. For example, some investigations have involved elective procedures with minimal blood loss, often reporting high correlation coefficients and low bias values that may present an overly optimistic view of the technology’s clinical utility [11,21]. However, such data may not be directly translatable to high-stakes surgical environments. The strength of our study lies in the deliberate selection of complex neurosurgical cases, such as craniosynostosis repair and spinal surgeries, where rapid hemodynamic shifts and substantial blood loss are the norms. Furthermore, approximately 70% of our patients were infants under 24 months; in this vulnerable group with low body weight, even small amounts of blood loss can have critical hemodynamic consequences and lead to rapid hemodilution, testing the limits of pulse co-oximetry in a way that low-risk surgeries or older cohorts cannot. By challenging the device with a mean blood loss of 15.9% of the estimated total blood volume and a 70.2% transfusion rate, our findings provide a more rigorous assessment of SpHb’s performance during the ‘worst-case’ intraoperative conditions.

In one of the few studies specifically targeting the pediatric neurosurgical population, Park et al. analyzed 119 paired samples from 20 patients and reported an overall mean bias of 0.90 ± 1.35 g/dL (95% LoA: −1.74 to 3.54 g/dL) with a correlation coefficient of r = 0.53 (*p* < 0.001) [22]. They also noted that the bias increased to 1.18 ± 1.28 g/dL specifically after volume resuscitation. While our study yielded a similar correlation (r = 0.567, *p* < 0.001), we observed a substantially higher systematic overestimation, with a mean bias of +1.60 ± 1.54 g/dL (LoA: −1.42 to 4.62 g/dL) across 210 paired measurements from 57 patients. The higher bias in our cohort is likely due to the age distribution; while Park et al. included children aged 10 months to 17 years, 70.2% of our patients were infants under 24 months. Furthermore, while both studies align on the device’s utility for trend-tracking (Park’s r = 0.75 for ΔHb vs. our 72.9% concordance rate), Park’s theoretical caution regarding transfusion decisions is directly substantiated by our finding of 0% sensitivity in detecting critical anemia (Hb < 8.0 g/dL).

Our findings significantly diverge from the accuracy metrics reported by Patino et al., who conducted a validation study on 158 data pairs and 105 delta pairs from 46 patients (aged 2 months to 17 years) [23]. While they reported a mean bias of 0.4 ± 1.3 g/dL (95% LoA: −2.2 to 3.0 g/dL), our study revealed a substantially higher systematic overestimation, with a bias of +1.60 ± 1.54 g/dL (LoA: −1.42 to 4.62 g/dL). Interestingly, the reference Hb range in our study (7.0–16.1 g/dL) was remarkably similar to that of Patino et al. (7.9–16.7 g/dL), yet the clinical performance of SpHb differed markedly. This discrepancy can be explained by the density of clinical events and demographic challenges in our cohort. First, the transfusion rate in our study was more than double that of Patino’s (70.2% vs. 33.0%), and our patients experienced a mean blood loss of 15.9% of estimated blood volume, indicating that our data points were more frequently captured during phases of acute, rapid hemodilution. Second, the age distribution played a pivotal role; whereas Patino et al. included a broad pediatric range up to 17 years, 70.2% of our patients were infants under 24 months, a group where smaller digit diameters and peripheral perfusion variability are known to compromise spectrophotometric accuracy. Although both studies align on the device’s trending capability—evidenced by Patino’s trending correlation of r = 0.76 and our concordance rate of 72.9%—our inclusion of deeper anemic states revealed a critical safety gap. While Patino et al. reported a 94% directional agreement within a relatively stable range, our findings in a more hemodynamically volatile infant cohort demonstrated a 0% sensitivity for detecting Hb < 8.0 g/dL, highlighting that SpHb may consistently overestimate oxygen-carrying capacity precisely when transfusion decisions are most imminent.

Regarding the clinical application of Pulse CO-Oximetry, Saracoglu et al. conducted a pivotal outcome-based study on 42 children undergoing fronto-orbital advancement, demonstrating that SpHb-guided management significantly optimized blood use [24]. They reported a substantial reduction in transfusion requirements (18.7% in the SpHb group vs. 50.0% in the control group; *p* = 0.038) and a lower mean volume of administered erythrocytes (4.4 ± 10.9 mL/kg vs. 13.5 ± 17.5 mL/kg; *p* = 0.03), which ultimately correlated with shorter ICU stays. While these findings initially suggest a clinical benefit, our detailed accuracy analysis provides a critical counter-perspective. The systematic overestimation observed in our study—a mean bias of +1.60 ± 1.54 g/dL—suggests that the lower transfusion rates reported by Saracoglu et al. might be confounded by the device’s tendency to report falsely high Hb levels. This concern is empirically supported by our finding of 0% sensitivity for detecting Hb < 8.0 g/dL and only 16.7% for Hb < 9.0 g/dL. Furthermore, with only 28.1% of our readings falling within 1.0 g/dL of the reference value, probably, the ‘avoided’ transfusions in outcome-focused studies may actually represent missed cases of critical anemia. Therefore, unlike Saracoglu et al., who advocate for SpHb as a primary clinical guide, we emphasize that in high-risk infant neurosurgery, SpHb should only serve as a ‘trigger for invasive blood sampling’ to prevent the life-threatening risks of masked anemia.

Unlike previous studies that primarily focused on Hb comparison, our study elucidates the physiological boundaries and ‘blind spots’ affecting SpHb performance. Our analysis demonstrates that the device’s accuracy is intrinsically linked to microcirculatory dynamics. Specifically, stratification of the PI revealed a paradoxical relationship: while higher perfusion levels (PI ≥ 5) significantly improved trend-tracking capability (r = 0.648), they simultaneously amplified the systematic error, with mean bias rising to 1.82 g/dL. Conversely, in the lowest perfusion category (PI < 0.3), although the mean bias appeared notably lower (0.65 g/dL), the correlation lost statistical significance (*p* = 0.138), effectively rendering the device unreliable despite the apparent numerical closeness. Beyond local perfusion deficits, systemic hemodynamic instability also compromised measurement quality; while the mean bias remained comparable to normotensive states during hypotensive episodes, the significant widening of the standard deviation (1.92 g/dL) indicates that low arterial pressure introduces erratic and unpredictable variability into the measurement algorithm. This hemodynamic volatility was further compounded by metabolic stress; in patients experiencing a significant drop in BE (>3 mEq/L from baseline), the device’s trend-tracking ability deteriorated (r dropped from 0.580 to 0.511). Most critically, in instances of severe acidosis (BE < −10 mEq/L), the systematic overestimation surged to +2.27 g/dL (vs. +1.56 g/dL in measurements without severe acidosis). This indicates that during profound metabolic failure, SpHb not only loses precision but substantially overestimates Hb levels. However, notably, this degradation occurred despite largely preserved tissue oxygenation, as lactate levels remained within physiological limits in 96.5% of the cohort (mean 1.66 mmol/L during hypotension). This dissociation suggests that SpHb failure is driven primarily by hemodynamic signal attenuation and acid-base shifts rather than tissue hypoxia. Regarding preoperative status, our findings challenged the conventional hypothesis that prolonged fasting compromises signal quality via dehydration-induced hypoperfusion. Contrary to expectations, patients fasting for >6 h exhibited superior precision (SD: 1.40 g/dL) and stronger correlation (r = 0.651) compared to those with shorter fasting durations (4–6 h; SD: 1.61 g/dL, r = 0.534). This indicates that the moderate dehydration associated with standard fasting protocols does not critically attenuate peripheral pulsatility; rather, the device performs more consistently in this group, potentially due to reduced fluid shifts before induction. Similarly, concerns that intraoperative fluid management would degrade optical readings were not supported. In the overall cohort, higher fluid intensity did not degrade performance. Even in the refined subgroup receiving solely crystalloids (N = 17), stratification by the subgroup median revealed nearly identical bias between low and high-volume recipients (+1.79 g/dL vs. +1.76 g/dL), confirming that hemodilution per se does not exacerbate measurement error.

Regarding ES administration, we observed a functional divergence where transfusion appeared to improve point-accuracy while potentially compromising trend reliability. Although post-transfusion values mathematically converged toward laboratory results (reducing mean bias from +1.83 to +1.28 g/dL), the correlation weakened (r decreased from 0.608 to 0.552). We hypothesize that this discrepancy might stem from two distinct mechanisms. The reduction in bias could be explained by the transfusion-induced rise in Hb to levels where the device tends to perform better, as suggested by our baseline stratification. Conversely, the degradation in correlation might indicate that SpHb could experience a lag during acute resuscitation, raising the possibility that the device fails to instantly mirror the rapid hematological shifts occurring in the central circulation before peripheral equilibration is fully complete.

Several limitations of our study should be acknowledged. First, the single-center design and the relatively small sample size, particularly within the specific subgroup stratifications (e.g., severe acidosis), may limit the generalizability of our findings to broader pediatric populations. Second, regarding reference methodology, we intentionally utilized ABG analysis as the primary comparator rather than solely relying on Co-oximetry. While laboratory analysis is the theoretical gold standard, ABG represents the ‘clinical gold standard’ in the dynamic environment of pediatric neurosurgery, where rapid decision-making is dictated by point-of-care testing. Therefore, our design reflects real-world operating room conditions rather than an artificial experimental setup. Moreover, unlike many studies limited to a single comparator, we strengthened our validation by incorporating a triple-comparison analysis (SpHb vs. ABG vs. Laboratory CBC) at the end of surgery, demonstrating that the device’s deviation pattern remained consistent across both reference methods. Third, we did not categorize patients based on skin pigmentation (Fitzpatrick skin type); although our study population was demographically homogeneous, skin tone remains a known variable in optical sensor physics that was not accounted for in this analysis. Fourth, although we identified significant deviations during physiological extremes, the absolute number of events with severe hypotension or profound acidosis was limited due to effective anesthetic management; therefore, future multicenter studies are needed to validate the device’s behavior in these rare, critical scenarios. Fifth, despite meticulous sensor placement, the potential influence of external pressure from surgical drapes or ambient light interference in the operating room environment could not be entirely excluded. Sixth, our study design was strictly limited to the intraoperative period to assess real-time diagnostic accuracy. Consequently, we did not evaluate long-term postoperative outcomes, such as intensive care unit length of stay, long-term morbidity, or the neurodevelopmental impacts of missed anemic events. Future outcome-based studies are required to determine how the intraoperative diagnostic limitations of SpHb identified in this study translate into long-term patient recovery. Seventh, multiple paired measurements from individual patients were analyzed as independent observations without adjusting for within-patient clustering, which may influence variance estimates in our agreement analyses.

## 5. Conclusions

This study delineates the performance variations across physiological states of SpHb monitoring in the high-stakes environment of pediatric neurosurgery. Our results indicate that SpHb is not a ‘one-size-fits-all’ replacement for invasive monitoring, but rather a context-dependent tool whose reliability fluctuates with microcirculatory dynamics. While the device demonstrates robust trend-tracking capability during stable perfusion and remains resilient against high-volume crystalloid hemodilution and prolonged fasting, it exhibits critical reduced diagnostic accuracy during physiological extremes. Specifically, the device tends to lose signal reliability during severe vasoconstriction (PI < 0.3) and substantially overestimates Hb levels during severe metabolic acidosis and high-perfusion states. Furthermore, the observed ‘lag effect’ during rapid blood transfusion suggests that SpHb may not instantly reflect acute hematological shifts. Therefore, in complex infant neurosurgery, SpHb should be positioned as a complementary ‘early warning’ trend monitor rather than a sole trigger for transfusion. The standard of care remains a hybrid approach: utilizing SpHb for continuous trend surveillance, while strictly validating any critical transfusion decision with invasive ‘gold standard’ methods—particularly when the patient is hypotensive, acidotic, or actively bleeding.

## Figures and Tables

**Table 1:** The demographic and perioperative characteristics of patients. Data are presented as mean ± SD (range or median) or number (percentage). ASA: American Society of Anesthesiologists; cm: centimeter; g: gram; dL: deciliter; min: minute; mL: milliliter; kg: kilogram; Hb: Hemoglobin; SD: Standard Deviation.

Parameter	Value (n = 57)
Demographics	
Age (months)	33.2 ± 43.8 (Median: 12, Range: 1–168)
Gender (Male/Female)	41 (71.9%)/16 (28.1%)
Weight (kg)	13.9 ± 10.2 (Range: 3.3–58.0)
Height (cm)	87.7 ± 28.5 (Range: 50.0–165.0)
ASA Physical Status	
ASA I	11 (19.3%)
ASA II	28 (49.1%)
ASA III	17 (29.8%)
ASA IV	1 (1.8%)
Preoperative Status	
Fasting Duration (hours)	6.8 ± 1.5 (Median: 6.0)
Baseline Hemoglobin (g/dL)	12.1 ± 1.5 (Range: 8.6–15.6)
Surgical Data	
Duration of Surgery (min)	198.6 ± 66.5 (Median: 200)
Total Blood Loss (mL)	147.6 ± 192.6 (Median: 100)
Total Blood Loss (mL/kg)	12.7 ± 15.5
Surgical Procedures	
Craniosynostosis Repair	30 (52.6%)
Intracranial Mass/Hemorrhage	14 (24.6%)
Kyphectomy	4 (7.0%)
Other	9 (15.8%)
Fluid & Transfusion Management	
Crystalloid Administration (mL/kg)	22.7 ± 13.0
Erythrocyte Suspension (ES) Transfusion	40 (70.2%)
Fresh Frozen Plasma (FFP) Transfusion	19 (33.3%)
Vasoactive Support Requirement	3 (5.3%)

**Table 2:** Correlation and Agreement Between SpHb and ABG Hb Stratified by Perfusion Index (PI). SD, standard deviation; g, gram; dL, deciliter.

PI Group	n	Pearson’s r	*p*-Value	Mean Bias (g/dL)	SD of Bias
PI < 0.3	11	0.477	0.138	0.65	1.70
0.3 ≤ PI < 1	45	0.637	<0.001	1.15	1.59
1 ≤ PI < 5	134	0.531	<0.001	1.80	1.50
PI ≥ 5	20	0.648	0.002	1.82	1.37

## Data Availability

The data presented in this study are available on request from the corresponding author due to privacy and ethical restrictions regarding pediatric patient data.

## Abbreviations

The following abbreviations are used in this manuscript:

Hb	Hemoglobin
SpHb	Continuous Non-invasive Hemoglobin
ABG	Arterial Blood Gas
PI	Perfusion Index
ASA	American Society of Anesthesiologists
ECG	Electrocardiography
SpO_2_	Pulse Oximetry Oxygen Saturation
NIBP	Non-invasive Blood Pressure
IV	Intravenous
TIVA	Total Intravenous Anesthesia
BIS	Bispectral Index
IABP	Invasive Arterial Blood Pressure
BMI	Body Mass Index
PaCO_2_	Partial Pressure of Arterial Carbon Dioxide
BE	Base Excess
CBC	Complete Blood Count
SD	Standard Deviation
LoA	Limits of Agreement
MAP	Mean Arterial Pressure
EBV	Estimated Blood Volume
ES	Erythrocyte Suspension
WHO	World Health Organization
PaO_2_	Partial Pressure of Arterial Oxygen
SaO_2_	Arterial Oxygen Saturation
g/dL	Grams per Deciliter
r	Pearson Correlation Coefficient
*p*	*p* value
